# Table of French Weights and Measures

**Published:** 1852-12

**Authors:** 


*Table of French Weights and Measures.*—The medical reader frequently meets, not only in original works, but in quoted extracts, and translations, the weights and measures peculiar to the French. For convenience of reference we copy the following table from the Canada Med. Journal:

| NEW MEASURES. | APPROXIMATE VALUE. | EXACT VALUE. | | | |
|---|---|---|---|---|---|
| | | ℔. | ℥. | ʒ. | grs. |
| 1 Centigramme. | 1-5 Grain. | 0 | 0 | 0 | 0.19 |
| 1 Decigramme. | 2 Grains. | 0 | 0 | 0 | 1.88 |
| 1 Gramme. | 20 Grains. | 0 | 0 | 0 | 18.82 |
| 10 Grammes. | 2 1-2 Gros. | 0 | 0 | 2 | 44.28 |
| 100 Grammes. | 3 Ounces 2 Gros. | 0 | 3 | 2 | 10.80 |
| 1 Kilogramme. | 2 Pounds. | 2 | 0 | 5 | 35.15 |

| OLD MEASURES. | | APPROXIMATE VALUE. | EXACT VALUE. | |
|---|---|---|---|---|
| 1 Grain. | gr. | 5 Centigrammes. | 0 Grammes. | ·033 |
| 1 Gross. | dram | 4 Grammes. | 3 | 82 |
| 1 Ounce. | oz. | 30 Grammes. | 30 | 59 |
| 1 Pound. | lb. | 500 Grammes. | 489 | 40 |

| | |
|---|---|
| Kilogramme, weight of 1 cubic decimetre of water of the temperature of 39 d. 12 m. Fahr. . . . . | 2.6803 ℔. troy.<br>2.1055 avoirdup. |
| Gramme, 1-1000th part of a kilogramme . . . | 1.5438 grs. troy.<br>0.9719 scruples.<br>0.032 oz. troy. |
| Decigramme, 1-00,000th of a kilogramme . . . | 1.5438 gr. troy. |
| Centigramme, 1-000,006th " . . . | 0.1543 gr. troy. |

